# Antidepressant Prevention of Postnatal Depression

**DOI:** 10.1371/journal.pmed.0030389

**Published:** 2006-10-03

**Authors:** Louise M Howard, Elizabeth Boath, Carol Henshaw

## Abstract

A systematic review sought to discover whether antidepressant drugs are effective in preventing postnatal depression. Only two small trials were found and it was not possible to draw firm conclusions.

Postnatal depression (PND) occurs in 10 to 15 percent of mothers [1] and is therefore the commonest complication of childbearing. The morbidity of PND for the mother and its potentially negative associations with neonatal and child development and on other family members are well established [2–4]. In theory, the antenatal period and early puerperium are opportune times to implement interventions to prevent PND because mothers are in frequent contact with health professionals. It is also increasingly recognised that some women who are diagnosed with depression postnatally have been depressed during the antenatal period [5,6], so it is important to detect and treat symptoms of depression antenatally to reduce the incidence of PND. We have therefore recently carried out a Cochrane review of antidepressant prevention of postnatal depression (see Text S1).

## The Review

We sought to discover whether antidepressant drugs are effective in the prevention of postnatal depression, to compare the effectiveness of different antidepressant drugs, with or without any other form of prevention for postnatal depression, i.e. hormonal treatment and psychological or social support, and to assess any adverse effects of antidepressant drugs in either the mother or the foetus/infant.

Criteria for inclusion were all published and unpublished randomised controlled trials of antidepressants to prevent postnatal depression in women who were pregnant or had given birth in the previous six weeks and who were not taking any antidepressant medication at the start of the trial. Women who had already been diagnosed with antenatal depression were excluded. Literature from 1945 to 2004 was methodically screened; this included a comprehensive literature search of electronic databases and was supplemented by hand searching and personal communication.

Two small studies fulfilled inclusion criteria for this review. No unpublished studies were identified. Both examined the effect of antidepressants initiated within 24 hours of delivery in women with a history of at least one episode of postnatal depression. The first study [7], with 51 participants, found that nortriptyline was no more effective than placebo in preventing a recurrence of postpartum major depressive disorder. The second trial [8] reported that sertraline was more effective than placebo in preventing a recurrence of postpartum major depression. Of fourteen patients who took sertraline, only one suffered a recurrence. In contrast, out of eight patients assigned to placebo, four (50%) suffered recurrences (*p* = 0.04), and the time before recurrence was longer in the sertraline-treated women compared with placebo-treated women (*p* = 0.012). Adverse effects found were headaches in two patients and hypomania in one patient, leading to withdrawal of all three patients. Dizziness and drowsiness were also reported significantly more often in the sertraline group.

A meta-analysis was not carried out because the two trials involved pharmacologically very different antidepressants.

## Implications of the Review

Both studies had serious methodological limitations, which means that no clear conclusions can be drawn about the effectiveness of antidepressants in preventing postnatal depression. The nortriptyline trial may have been statistically underpowered, and less than half the women eligible for the trial took part. The second small trial found some evidence that sertraline was effective in preventing postnatal depression, but only 25 women were recruited in total and the data were not subjected to an intention-to-treat analysis. This randomised controlled trial only included white married women of middle to high socioeconomic status and the nortriptyline trial did not give details of sociodemographic characteristics; the women were therefore not representative of women at risk of PND. The studies also excluded women taking antidepressants in the first trimester. Both trials included breast-feeding women but did not give details of any adverse effects on the infants and did not follow up infants exposed to the antidepressants to examine neurodevelopmental outcomes in the long term.

The group of women at highest risk of PND because of a recent history of a major depressive episode [5] may take antidepressants at conception as part of relapse prevention. Continuation of antidepressants through pregnancy in such women may prevent postnatal depression, but this was not tested in these studies. Antidepressants may also be more likely to prevent postnatal depression if medication is used which successfully treated previous episodes of depression. In addition, the trials did not compare antidepressant therapy with any other form of treatment, and both trials had very short periods of follow-up time with no assessment of the impact on the infant. The paucity of trials in this area and the small size of the trials published may reflect a reluctance to recruit women in the postpartum period into antidepressant trials, as there is evidence from studies of treatment of PND that women are often unwilling to take antidepressants [9,10]. There is particular concern regarding prescribing antidepressants when women are breast-feeding, as there is limited evidence on the extent to which infants are exposed to antidepressants through breast milk and the long-term effect this may have on the infant [11]. The evidence therefore does not allow us to make any recommendations about the role of antidepressants in preventing postpartum depression.

Future research should refine the identification of high-risk women, compare the effectiveness of antidepressants and psychosocial treatments for women with depression in the postnatal period, and carry out long-term follow-up of women and their children, including monitoring of adverse effects for the mother and infant.

At present, although there have been many trials of psychological and psychosocial interventions to prevent PND, there is little evidence of effectiveness for these interventions [12,13]. This may partly reflect the problem of identifying women at high risk of PND, and several studies have therefore targeted all women, either during pregnancy or in the early postnatal period. However, a systematic review of preventative interventions for PND found that identifying women “at risk” assisted in the prevention of postnatal depression. In addition, postnatal interventions were found to be more effective than antenatal interventions, and individually based interventions were more effective than group interventions [12]. Guidelines based on a review of the available evidence have concluded that in high-risk women it may be effective to provide postnatal visits, interpersonal therapy, and/or educational programmes on preparation for parenthood [13]. As the prescription of antidepressants is difficult in the postnatal period, for the reasons detailed above, the current evidence base therefore supports offering women at risk of PND intensive professionally based postpartum support for prevention of PND.

## Supporting Information

Text S1 Cochrane Review Howard LM, Hoffbrand S, Henshaw C, Boath L, Bradley E (2005) Antidepressant prevention of postnatal depression. Cochrane Database Syst Rev 2: CD004363.
This data supplement can be freely accessed on the *PLoS Medicine* Web site, but it is not published under the Creative Commons Attribution License.
Copyright © 2006 The Cochrane Collaboration. Published by John Wiley and Sons. All rights reserved.
(168 KB PDF).

## Abbreviations

PND: postnatal depression
