# Supplementary material for: Antidepressant Prevention of Postnatal Depression
Source: PLoS Med. 2006 Oct 3;3(10):e389. doi: 10.1371/journal.pmed.0030389 (PMC1590037; doi:10.1371/journal.pmed.0030389)
Supplement: Supplementary file 1 [file pmed.0030389.sd001.pdf]

Antidepressant prevention of postnatal depression (Review)

Howard LM, Hoffbrand S, Henshaw C, Boath L, Bradley E

This is a reprint of a Cochrane review, prepared and maintained by The Cochrane Collaboration and published in *The Cochrane Library* 2006, Issue 2

http://www.thecochranelibrary.com

Copyright © 2006 The Cochrane Collaboration. Published by John Wiley & Sons, Ltd

TABLE OF CONTENTS

ABSTRACT . . . 1
PLAIN LANGUAGE SUMMARY . . . 2
BACKGROUND . . . 2
OBJECTIVES . . . 2
CRITERIA FOR CONSIDERING STUDIES FOR THIS REVIEW . . . 3
SEARCH METHODS FOR IDENTIFICATION OF STUDIES . . . 3
METHODS OF THE REVIEW . . . 4
DESCRIPTION OF STUDIES . . . 5
METHODOLOGICAL QUALITY . . . 5
RESULTS . . . 5
DISCUSSION . . . 5
AUTHORS' CONCLUSIONS . . . 6
POTENTIAL CONFLICT OF INTEREST . . . 6
ACKNOWLEDGEMENTS . . . 6
SOURCES OF SUPPORT . . . 6
REFERENCES . . . 6
TABLES . . . 8
    Characteristics of included studies . . . 8
ANALYSES . . . 9
    Comparison 01. Nortriptyline versus placebo . . . 9
    Comparison 02. Sertraline versus placebo . . . 9
INDEX TERMS . . . 9
COVER SHEET . . . 9
GRAPHS AND OTHER TABLES . . . 10
    Analysis 01.01. Comparison 01 Nortriptyline versus placebo, Outcome 01 Recurrence of postpartum MDD . . . 10
    Analysis 02.01. Comparison 02 Sertraline versus placebo, Outcome 01 Recurrence of postpartum MDD . . . 11

Copyright © 2006 The Cochrane Collaboration. Published by John Wiley & Sons, Ltd

Antidepressant prevention of postnatal depression (Review)

Howard LM, Hoffbrand S, Henshaw C, Boath L, Bradley E

This record should be cited as:
Howard LM, Hoffbrand S, Henshaw C, Boath L, Bradley E. Antidepressant prevention of postnatal depression. *The Cochrane Database of Systematic Reviews* 2005, Issue 2. Art. No.: CD004363.pub2. DOI: 10.1002/14651858.CD004363.pub2.

This version first published online: 20 April 2005 in Issue 2, 2005.
Date of most recent substantive amendment: 31 January 2005

ABSTRACT

Background
Postnatal depression is a common and important complication of childbearing. Untreated depression can lead to potentially negative effects on the foetus and infant, in addition to serious morbidity for the mother. The use of antidepressants during pregnancy for prevention of postnatal depression is unclear, due to the possibility of adverse effects on the mother and developing foetus, and the difficulty of reliably identifying the women who would go on to develop postnatal depression.

Objectives
To evaluate the effectiveness of different antidepressant drugs in addition to standard clinical care in the prevention of postnatal depression.
To compare the effectiveness of different antidepressant drugs and with any other form of intervention for postnatal depression i.e. hormonal, psychological or social support.
To assess any adverse effects of antidepressant drugs in either the mother or the foetus/infant.

Search strategy
The register of clinical trials maintained and updated by the Cochrane Depression, Anxiety and Neurosis Group and the Cochrane Pregnancy and Childbirth Group.

Selection criteria
Randomised studies of antidepressants alone or in combination with another treatment, compared with placebo or a psychosocial intervention in non-depressed pregnant women or women who had given birth in the previous six weeks (i.e. women at risk of postnatal depression)

Data collection and analysis
Data were extracted independently from the trial reports by the authors. Missing information was requested from investigators wherever possible. Data were sought to allow an "intention to treat" analysis.

Main results
Two trials fulfilled the inclusion criteria for this review. Both looked at women with a past history of postpartum depression. Nortriptyline (n=26) (Wisner 2001) did not show any benefit over placebo (n=25). Sertraline (n=14) Wisner 2004 reduced the recurrence of postnatal depression and the time to recurrence when compared with placebo (n=8). Intention to treat analyses were not carried out in either trial.

Authors' conclusions
It is not possible to draw any clear conclusions about the effectiveness of antidepressants given immediately postpartum in preventing postnatal depression and, therefore, cannot be recommended for prophylaxis of postnatal depression, due to the lack of clear evidence. Larger trials are needed which also include comparisons of antidepressant drugs with other prophylactic treatments to reflect clinical practice, and examine adverse effects for the foetus and infant, as well as assess womens' attitudes to the use of antidepressants at this time.

Copyright © 2006 The Cochrane Collaboration. Published by John Wiley & Sons, Ltd

PLAIN LANGUAGE SUMMARY

Postnatal depression is a common and important disorder with negative implications for the mother, the infant and the wider family. Women who are not depressed, but at high risk of postnatal depression, such as those with a previous history of a postpartum mood disorder, may wish to consider antidepressant prevention during pregnancy or early postpartum. This review addresses the effectiveness of such treatment. Only two small trials met the criteria for inclusion. Both trials used medication immediately postpartum. The drugs were nortriptyline, a tricylic antidepressant (TCA) and sertraline, a selective serotonin reuptake inhibitor (SSRI). Both drugs were compared only to placebo. Nortripyline was not shown to have any benefit over placebo; there was some evidence that sertraline was effective both in reducing the incidence of recurrent postpartum depression and in increasing the time to recurrence. However, both trials involved only very small numbers of women and did not use intention to treat analyses. There is, therefore, no clear evidence for the use of these antidepressants in the prevention of postnatal depression.

BACKGROUND

Postnatal depression (PND) occurs in 10-15% of mothers (O'Hara 1996), and is therefore the commonest complication of childbearing. The morbidity of the illness for the mother and its potentially negative effects on neonatal and child development and on other family members are well established (Cox 1982, Cooper 1998, O'Hara 1990). The antenatal period and early puerperium are theoretically times of opportunity for prevention of PND because of frequent contact with health professionals. It is also increasingly recognised that some women who become depressed postnatally have, in retrospect, been depressed during the antenatal period (Kumar 1984, Evans 2001), so it is important to detect and treat symptoms of depression antenatally to reduce the incidence of PND. Women with antenatal depression are not necessarily the same cohort of women who develop postnatal depression.

Prevention strategies can be offered to the whole population potentially at risk, or offered only to those at higher risk, eg those with a previous postnatal depression, who may be more motivated to accept preventative interventions, and may be more likely to benefit from them. Targeting high risk groups may not have much impact on the incidence of PND in the population (Rose 1985), but this may be the only realistic approach, particularly when considering potential adverse effects of antidepressant prophylaxis, which may need to be started during pregnancy, and could, therefore, have an effect on the fetus, in addition to the mother. However, identifying factors for women at high risk of PND has proved disappointing (Appleby 1994, Austin 2002), with low population attributable fractions for independent risk factors such as unplanned pregnancy and unemployment in mother and/or head of the household (0.14 and 0.24/0.25 respectively (Warner 1996). Cooper 1996 has validated a predictive index for use in pregnant women, but only 35% of high risk women identified using this index would be expected to become depressed in the context of primary care. O'Hara 1996, in a meta-analysis, found that a past history of psychopathology produces the most consistent and largest effect size. However, of those identified as at high risk, including those with a history of bipolar disorder, the majority will not become depressed and pharmacological prevention for all at high risk may not be justified. Decisions concerning the use of prophylactic medications during pregnancy must consider the potential effect on the foetus. Data on immediate risks to the foetus from antidepressants are limited, and little is known about the potential long term risks. However, there is no evidence that the commonly used tricyclic antidepressants (TCAs) and selective serotonin reuptake inhibitors (SSRIs) are teratogenic, though these data are from small studies and adverse drug reports to teratology information services (Heath 2001). Neonatal withdrawal symptoms have been reported in babies born to mothers taking antidepressants (Costei 2002, Hendrick 2003, Laine 2003, Zeskind 2004, Kallen 2004), though the incidence of such complications is unknown (Watson 1984, Heath 2001).

Prophylaxis could alternatively begin postpartum, but then issues regarding breastfeeding need to be considered. There are little data on the safety of breastfeeding for the infant when the nursing mother takes antidepressants (Hoffbrand 2001). The findings to date suggest that the benefits of breastfeeding and taking antidepressants outweigh the risks in women who need treatment for depression (Yoshida 1999). The risk-benefit equation is much less clear for women offered prophylactic antidepressant treatment postpartum.

In view of the adverse effects of medication, psychosocial preventative interventions may be more appropriate in the prevention of PND. Other Cochrane reviews examine the effect of psychosocial interventions (Dennis 2002) and hormonal prophylaxis (Lawrie 2002), and, therefore, these are not covered in this review. However, the effects of antidepressants compared to, or in combination with, psychosocial interventions as prophylaxis for postnatal depression are reviewed here.

OBJECTIVES

1. To evaluate the effectiveness of antidepressant drugs in addition to standard clinical care in the prevention of postnatal depression.

Copyright © 2006 The Cochrane Collaboration. Published by John Wiley & Sons, Ltd

2. To compare the effectiveness of different antidepressant drugs and with any other form of prevention for postnatal depression i.e. hormonal, psychological or social support.

3. To assess any adverse effects of antidepressant drugs in either the mother or the foetus/infant.

CRITERIA FOR CONSIDERING STUDIES FOR THIS REVIEW

Types of studies

All published and unpublished randomised controlled trials.

Types of participants

Women who are pregnant or have given birth in the last six weeks, who were not taking any antidepressant medication at the start of the trial. Trials may include only women with a history of depression and/or postnatal depression, or offer the intervention to all pregnant women but women must not be depressed at the beginning of the trial. Women who already have an antenatal depression were excluded.

Types of intervention

Any type of antidepressant medication at any dose alone or in combination with another treatment initiated in at least one arm of a trial compared with any other treatment, or placebo, or standard clinical care.

Types of outcome measures

1. A very broad definition of postnatal depression was used that ignored the timing and onset of depression to include all women who became depressed during the first six months postpartum. This included an estimate of depression as measured by the investigators using any of the following: use of screening instrument, for example, the Edinburgh Postnatal Depression Scale (EPDS) (Cox 1987), use of standard observer rated depression symptom scales, by a recognised diagnostic scheme e.g. Diagnostic and Statistical Manual of Mental Disorders (DSM IV) or the International Classification of Disease (ICD10), or by other standardised criteria, for example, the Research Diagnostic Criteria (RDC); (Spitzer 1978). The threshold scores used for the respective scales were those used by the investigators in the trials.

2. Adverse events experienced by mother and/or foetus or nursing baby.

3. Acceptability of treatment both as assessed directly by questioning trial participants and indirectly by the drop-out rates.

4. Cognitive and emotional development of the infant/ child.

Information was sought about other outcomes:
5. Overall maternal satisfaction.
6. Improvement in the maternal relationship with the baby.
7. Improvement in the ability of the mother to carry out daily activities and in her social functioning.
8. The establishment or continuation of breastfeeding.
9. Prevention of neglect or abuse of the baby.
10.The effect on marital and family relationships.

SEARCH METHODS FOR IDENTIFICATION OF STUDIES

See: Depression, Anxiety and Neurosis Group methods used in reviews.

Electronic Searches:
The Cochrane Collaboration Depression Anxiety and Neurosis group trials register (CCDAN TR-Studies). This is a specialised register that contains more than 7,000 records on trials comparing treatment options that are within the scope of the CCDAN. The register is updated regularly adding the results on searches of The Cochrane Library, CINAHL, EMBASE, LILACS, MEDLINE, National Research Register, PSYCLIT, PSYCINFO, PSYNDEX and SIGLE. Also, quarterly systematic screening of relevant journals and conference proceedings takes place (for information on the full search strategies, visit http://web1.iop.kcl.ac.uk/IoP/ccdan/index.htm.)

The search was completed in July 2004.

a/ CCDANCTR was searched using the terms "Depression, Postpartum"

b/ The Cochrane Central Register of Controlled Trials (CENTRAL) and the specialized register of the Cochrane Pregnancy and Childbirth Group were searched with the following terms
Postnatal or postpartum or puerper*

c/ The HSRProj in the National Library of Medicine, Washington D.C was checked for unpublished trials.

d/ References from MIDIRS Midwifery Database was searched.

e/ Current Controlled Trials web site: http://www.controlled trials.com/terms2.cfm was examined for ongoing studies

f/ The Science Citation Index was checked for references to all included studies.

Handsearches:
a/ The reference lists of identified studies was checked.

b/ Relevant book chapters and their bibliographies were searched (details available from authors on request).

c/ Conference proceedings were examined where possible (details available from authors on request).

Personal Communication:

Copyright © 2006 The Cochrane Collaboration. Published by John Wiley & Sons, Ltd

a/ Pharmaceutical companies were contacted directly for any relevant unpublished data

b/ Contact was made with authors of identified trials and with experts in the field including a search for non-English material. (Professor L Appleby, Professor P Boyce, Dr T Brugha, Dr L Cohen, Dr N Glangeaud, Dr S Glasser, Dr M Marks, Dr A Wieck, Professor K Wisner)

c/ Contact with the Marcé Society through the newsletter was made

d/ Self help groups were contacted (the National Childbirth Trust (NCT), The Association for Postnatal Illness, Postnatal Distress Association of Ireland, Postpartum Support International (PSI) and PaNDA)

METHODS OF THE REVIEW

Study selection:
Abstracts of studies identified in the above search were examined by three authors. The full article was obtained for any publication which was potentially relevant. Trials under consideration were assessed for whether they fulfilled the inclusion criteria and methodological quality without regard to their results. Where the authors disagreed, the matter was discussed with the other authors until agreement was reached.

Assessment of Methodological Quality:
The methodological quality of the selected trials was assessed by three independent authors (CH, EB & EB). Details of randomisation, concealment of allocation, blinding and exclusion analyses were recorded and evaluated. Unbiased methods of randomisation considered acceptable included random numbers generated by computer or sequentially numbered opaque sealed envelopes containing random allocation. A rating was assigned to each trial, based on the quality rating system developed by the Cochrane Collaboration Depression, Anxiety and Neurosis Group and the categories described in the Cochrane Collaboration Handbook. Only categories A or B were to be included in meta-analyses. Data from other studies were to be described in the 'excluded studies' tables. Where the three authors disagreed, the matter was to be discussed with the other authors until clear agreement is reached and if necessary further information sought from the trial investigator(s). Reasons for exclusion of any apparently eligible trial were to be clearly described. Tables were to be used to display characteristics of eligible trials, including those that were excluded, with the reasons for exclusion.

Data collection:
Data were extracted independently from the trial reports by the authors. A data extraction form was designed and piloted specifically for the review. Missing information was requested from investigators wherever possible. Where a dispute arose, it was resolved by discussion between the authors. When this was not possible, further information was sought from the trial investigator(s). All exclusion/dropouts were identified. If no information was available (from the report or investigator), it was assumed that dropout was because of side effects or treatment failure. Where possible, data were sought to allow an 'intention-to-treat' analysis. Data were entered into Review Manager 4.2 software (RevMan) by one author (SH) and all entries were checked by the other author (LH).

Data synthesis:
Trials using different treatments were analysed separately, and the results combined only if there was no reason to think that they differed in relevant ways. A variety of depression rating scales have been used; only scales published in peer reviewed journals were used for data extraction. Where possible, direct comparisons were made between trials using the same rating scales. When the subjects were assessed using more than one rating scale, all data were presented.

Statistical analyses used the RevMan software. The relative risk and 95% confidence intervals were calculated for results using categorical data. Continuous data were pooled as weighted mean differences. Meta-analytic methods for continuous data assumed that the underlying distribution of the measurements were normal. The ratio of the mean to its standard deviation gives a crude way of assessing skew; if the ratio were less than 1.65 for any group in a trial, the individual results (if available from investigators) were log transformed and if this achieved a normal distribution, the mean was obtained before being included in an analysis with similarly log transformed results. If this was not possible then the skewed data were presented descriptively only.

When overall results were significant, the number needed to treat (NNT) to prevent one woman developing PND was calculated. Separate NNT calculations were carried out for high risk and low risk groups, as relative risk reduction can vary across different baseline risk (Smeeth 1999). Different trials use different definitions of high-risk groups, but as the most predictive risk factor for postnatal depression is a past history of psychopathology (O'Hara 1996), any trial that includes a history of depression in its definition of its high-risk group was included for the NNT calculation for a high-risk group. Other trials offered prophylaxis to all childbearing women, which is here defined as a low risk group.

Heterogeneity between trial results is investigated and in the event of significant statistical heterogeneity, the results are not combined in a meta-analysis. As a test of robustness of our results, a sensitivity analysis is conducted to assess the effects of excluding lower quality studies.

An intention to treat analysis is carried out where possible for dichotomous data. When data on drop-outs are carried forward and included in the efficacy evaluation (Last Observation Carried

Copyright © 2006 The Cochrane Collaboration. Published by John Wiley & Sons, Ltd

Forward, LOCF), they are analysed according to the primary studies. Where this is not possible, end-point data for trial completers are used. We assume that for incomplete continuous data, intention-to-treat analyses would be impossible. In these cases we analyse data as they were presented in the original publications. Sub-group analyses are carried out, if possible, to examine the effect in women with no history of depression versus those with a history of depression, and women started on antidepressants during pregnancy (and if numbers permit, third trimester versus earlier) versus those started postpartum. In addition relevant narrative from participants is included as qualitative commentary, where possible.

DESCRIPTION OF STUDIES

Two studies fulfilled inclusion criteria for this review. No unpublished studies were identified. Details are provided in the characteristics of included studies table.

Wisner 2001 - a double blind randomised controlled trial comparing nortriptyline and placebo, initiated within 24 hours of delivery in women with a history of at least one episode of postpartum major depression.

Wisner 2004 - a double blind randomised controlled trial comparing sertraline and placebo, initiated within 24 hours of delivery where possible in women with a history of at least one episode of postpartum depression.

METHODOLOGICAL QUALITY

The Wisner 2001 study was conducted double blind, and the integrity of the blind was tested - relevant staff did not successfully identify drug assignment more often than by chance. However, one side effect (constipation) was more prominent in patients on antidepressants (p=0.01), indicating the possibility that residual unblinding effects may have occurred. Random allocation was used, though details of the randomisation were not given. An intention to treat analysis was not carried out - the four patients who declined to take the study drug after randomisation and the one patient who developed mania were not included in the analysis.

The Wisner 2004 study was also conducted double blind, and the integrity of the blind was tested and found to be successfully hidden. Randomisation was used, but details are not given. An intention to treat analysis was not carried out - three patients allocated to sertraline refused to participate, and were not included in the analysis.

RESULTS

Wisner 2001 reported that nortriptyline was no more effective than placebo in preventing a recurrence of postpartum major depressive disorder. There was no difference in the rate of recurrence (Fisher's exact test p = 1) or the time to recurrence (exact log rank <0.001, exact p =0.83 in each group). There was also no difference in the time to recurrence between the two groups when stratified by presence of non-postpartum depressive episodes (exact log-rank < 0.001, exact p = 0.83) (see characteristics of included studies table.) Five subjects who took nortriptyline were defined as non-compliant (serum nortriptyline level <50 ng/mL). Censoring of these subjects at the time of noncompliance did not change the results of the recurrence analysis (exact log rank <0.001, p =0.83). The only adverse effect found was constipation, which was significantly more frequent in women taking nortiptyline (78%) compared with placebo (22%) (Fischer's exact test p = 0.001).

Wisner 2004 reported that sertraline was more effective than placebo in preventing a recurrence of postpartum major depression. Of fourteen subjects who took sertraline one suffered a recurrence; of 8 (70.1%) assigned to placebo, 4 (50%) suffered recurrences (p=0.04). Two of the nine women who completed the trial on sertraline became depressed as the drug was tapered (week 20) or shortly after discontinuation (week 26). The time to recurrence was longer in the sertraline-treated women compared with placebo-treated women (p=0.012). The women in the study were compliant, as evidenced by maternal serum sertraline levels. Adverse effects found were headaches in two patients leading to rapid withdrawal. One subject was removed due to hypomania, dizziness (50% in sertraline compared with 13% in placebo, p=0.042) and drowsiness (100% sertraline, 50% placebo, p=0.012).

A meta-analysis was not carried out because the two trials involved pharmacologically very different antidepressants and, as SSRIs may be effective in menstrually related mood symptoms, whereas tricyclic antidepressants such as nortriptyline are not, we decided that sertraline and nortriptripyline are too clinically heretogeneous to combine in a meta- analysis.

DISCUSSION

Only two eligible trials of antidepressants for the prevention of postnatal depression were identified. The first provided no evidence for the effectiveness of nortriptyline in preventing recurrence of postnatal depression, though the trial may have been underpowered. Less than half the women eligible for the trial took part, possibly because pregnant and nursing women do not want to take medication. A study of antidepressants for the treatment of postnatal depression had difficulty recruiting for this reason (Hoffbrand 2001). Antidepressants may be more likely to prevent postnatal depression if medication which successfully treated previous episodes of depression is used (rather than nortriptyline for

Copyright © 2006 The Cochrane Collaboration. Published by John Wiley & Sons, Ltd

all women, as in this study). However, the second small trial found some evidence that sertraline was effective in preventing postnatal depression, though this trial has a number of methodological difficulties: only 25 women were recruited, the analysis was not intention to treat and the ratio of active drug to placebo was 2:1. It is also worth noting that in this trial the women were recruited from a psychiatric outpatient setting, suggesting that they may have been very unwell previously, and so unusually motivated to take an antidepressant. Wisner 2004 suggests the positive result may be due to SSRIs increasing brain levels of neuroactive steroids, which may explain why sertraline may lower the risk of depression in the postpartum milieu (Griffin 1999). This trial clearly needs repeating with a larger sample size.

These studies excluded women taking antidepressants in the first trimester. The group of women at highest risk of postpartum depression because of a recent history of a major depressive episode (Kumar 1984) may be taking antidepressants at conception as part of relapse prevention. A recent systematic review found that continuing antidepressant treatment after a depressive episode reduces the odds of relapse by 70% (95% CI 62-78) compared with treatment discontinuation (Geddes 2003). Continuation of antidepressants through pregnancy in such women may prevent postnatal depression, but this was not tested in these studies.

The trials did not look at antidepressant therapy compared with any other form of treatment, and both trials to date have had very short periods of follow-up time, with no assessment of the impact on the infant. We were therefore only able to address the first of our three objectives.

AUTHORS' CONCLUSIONS

Implications for practice

This systematic review found only two studies of antidepressant prophylaxis of postnatal depression. Nortriptyline was not significantly more effective at preventing postnatal depression than placebo, but one small study found sertraline was significantly more effective than placebo at preventing postnatal depression. It is not possible from these two studies to draw any clear conclusions about the effectiveness of antidepressants in preventing postnatal depression. Furthermore, there has been no research into starting antidepressant prophylaxis during pregnancy. Therefore, the evidence does not allow us to make any recommendations about the role of antidepressants in preventing postpartum depression.

Implications for research

A cost benefit analysis of antidepressants for women at high risk of postnatal depression is not possible without further research in this area. This should include further refinement of the identification of high risk women, comparisons of the effectiveness of antidepressants and psychosocial treatments for women with depression in the postnatal period, and long term follow-up of women and their children, including monitoring of adverse effects for the mother and infant. Future studies should also investigate womens' attitudes to the use of antidepressants at this time.

POTENTIAL CONFLICT OF INTEREST

None

ACKNOWLEDGEMENTS

The authors would like to thank the Cochrane Collaboration Depression Anxiety and Neurosis Group for their advice with this review.

SOURCES OF SUPPORT

External sources of support

- No sources of support supplied

Internal sources of support

- Health Services Research Dept, Institute of Psychiatry UK

REFERENCES

References to studies included in this review

Wisner 2001 *{published data only}*

* Wisner KL, Perel JM, Peindl KS, Hanusa BH, Findling RL, Rapport D. Prevention of recurrent postpartum depression: a randomized clinical trial. *Journal of Clinical Psychiatry* 2001;**62**(2):82–6.

Wisner 2004 *{published data only}*

* Wisner KL, Perel JM, Peindl KS, Hanusa BH, Piontek CM, Findling RL. Prevention of postpartum depression: a pilot randomized clinical trial. *American Journal of Psychiatry* 2004;**62**(2):82–6.

Additional references

Appleby 1994

Appleby L, Gregoire A, Platz C, Martin P, Kumar R. Screening women for high risk of postnatal depression. *Journal of Psychosomatic Research* 1994;**38**:539–45.

Austin 2002

Austin MP, Lumley J. Antenatal screening for women at risk of postnatal depression. *Acta Psychiatrica Scandinavica* 2002;**106**:1–8.

Copyright © 2006 The Cochrane Collaboration. Published by John Wiley & Sons, Ltd

Cooper 1996
Cooper PJ, Murray L, Hooper R, West A. The development and validation of a predictive index for postpartum depression. *Psychological Medicine* 1996;**26**(3):627–34.

Cooper 1998
Cooper PJ, Murray L. Postnatal depression. *BMJ* 1998;**316**(7148): 1884–6.

Costei 2002
Costei A, Kozer E, Ho T, Ito S, Koren G. Perinatal outcome following third trimester exposure to paroxetine. *Archives of Pediatrics and Adolescent Medicine* 2002;**156**:1129–32.

Cox 1982
Cox J, Connor Y, Kendell. Prospective study of the psychiatric disorders of childbirth. *British Journal of Psychiatry* 1982;**140**:111–7..

Cox 1987
Cox JL, Holden J, Sagovsky R. Detection of postnatal depression: development of the 10-item Edinburgh Postnatal Depression Scale (EPDS). *British Journal of Psychiatry* 1987;**150**:782–6.

Dennis 2002
Dennis CL, Kavanagh J. Psychosocial interventions for preventing postpartum depression. In: *The Cochrane Library*, 4, 2002.. Oxford: Update Software.

Evans 2001
Evans J, Heron J, Francomb H, Oke S, Golding J. Cohort study of depressed mood during pregnancy and after childbirth. *BMJ* 2001; **323**(7307):257–60.

Geddes 2003
Geddes JR, Carney SM, Davies C, Furukawa TA, Kupfer DJ, Frank E, et al. Relapse prevention with antidepressant drug treatment in depressive disorders: a systematic review. *Lancet* 2003;**361**:653–51.

Griffin 1999
Griffin LS, Mellon SH. Selective serotonin reuptake inhibitors directly alter activity of neurosteroidogenic enzymes. *Proceedings of the National Academy of Sciences* 1999;**96**(23):13512–7.

Heath 2001
Heath AC, Yonkers KA. *Management of Psychiatric Disorders in Pregnancy*. London: Arnold, 2001.

Hendrick 2003
Hendrick V, Stowe ZN, Altshuler L, Hwang S, Lee E, Haynes D. Placental passage of antidepressant medication. *American Journal of Psychiatry* 2003;**160**(5):993–6.

Hoffbrand 2001
Hoffbrand S, Howard L, Crawley H. Anti-depressant drug treatment for postnatal depression. In: *The Cochrane Library*, 2, 2001. Oxford: Update Software.

Kallen 2004
Kallen B. Neonate characteristics after maternal use of antidepressants in late pregnancy. *Archives Pediatric Adolescent Medicine* 2004;**158** (4):312–6.

Kumar 1984
Kumar R, Robson K. A prospective study of emotional disorders in child bearing women. *British Journal of Psychiatry* 1984;**144**:35–47.

Laine 2003
Laine K, Heikkinen T, Ekblad U, Kero P. Effects of exposure to selective serotonin reuptake inhibitors during pregnancy on serotonergic symptoms in newborns and cord blood monoamine and prolactin concentrations. *Archives of General Psychiatry* 2003;**60**(7):720–6.

Lawrie 2002
Lawrie TA, Herxheimer A, Dalton K. Oestrogens and progestogens for preventing and treating postnatal depression. In: *The Cochrane Library*, 4, 2002. Oxford: Update Software.

Murray 1996
Murray L, Cooper PJ. The impact of postpartum depression on child development. *International Review of Psychiatry* 1996;**8**(1):55–63.

O'Hara 1990
O'Hara M, Zekoski EM, Philipps LH, Wright EJ. Controlled prospective study of postpartum mood disorders: comparison of childbearing and nonchildbearing women. *Journal of Abnormal Psychology* 1990;**99**(1):3–15.

O'Hara 1996
O'Hara M, Swain AM. Rates and risk of postpartum depression - a meta-analysis. *International Review of Psychiatry* 1996;**8**(1):37–54.

Rose 1985
Rose G. Sick individuals and sick populations. *International Journal of Epidemiology* 1985;**14**(1):32–9.

Smeeth 1999
Smeeth L, Haines A, Ebrahim S. Numbers needed to treat derived from meta-analyses - sometimes informative, usually misleading. *BMJ* 1999;**318**(7197):1548–51.

Spitzer 1978
Spitzer R, Endicott J, Robins E. Research Diagnostic Criteria; rationale and reliability. *Archives of General Psychiatry* 1978;**35**:773–82.

Warner 1996
Warner R, Appleby L, Whitton A, Faragher B. Demographic and obstetric risk factors for postnatal psychiatric morbidity. *British Journal of Psychiatry* 1996;**168**:607–11.

Watson 1984
Watson JP, Elliot SA, Rugg AJ, Brough DI. Psychiatric disorder in pregnancy and the first postnatal year. *British Journal of Psychiatry* 1984;**144**:453–62.

Yoshida 1999
Yoshida K, Smith B, Kumar R. Psychotropic drugs in mothers' milk: a comprehensive review of assay methods, pharmakokinetics and safety of breastfeeding. *Journal of Psychopharmacology* 1999;**139**:64–80.

Zeskind 2004
Zeskind PS, Stephens LE. Maternal selective serotonin reuptake inhibitor use during pregnancy and newborn neurobehaviour. *Pediatrics* 2004;**113**(2):368–75.

Zuckerman 1990
Zuckerman B, Bauchner H, Parker S, Cabral H. Maternal depressive symptoms during pregnancy and newborn irritability. *Developmental and Behavioural Pediatrics* 1990;**11**(4):190–4.

* *Indicates the major publication for the study*

Copyright © 2006 The Cochrane Collaboration. Published by John Wiley & Sons, Ltd

TABLES

Characteristics of included studies

Study	Wisner 2001
Methods	Randomised controlled trial. Method of randomisation not described.<br>121 women eligible, 69 refused to participate, 5 withdrew after randomisation (1 developed mania in 1st week postpartum and was withdrawn, 4 refused medication.)<br>56 women randomised by strata (past history of only postpartum major depressive disorder vs history of post partum major depressive disorder and non postpartum major depressive disorder) : 26 to nortriptyline, 25 to placebo (same number of capsules).<br>Outcome assessed by blind assessor.
Participants	Recruited from maternity hospital, USA. Inclusion criteria: women up to 6 months postpartum, < or = 35 weeks gestation, aged 21-45 years, history of at least 1 episode of postpartum major depressive disorder meeting Research Diagnostic Criteria for major depression and Hamilton Rating Scale for Depression (HAM - D) score > 14 within 3 months of a live birth, within 5 years of study enrolment.<br>Exclusion criteria - history of psychotic illness, antidepressants within 1st trimester, chronic depression, other axis 1 diagnosis except generalised anxiety disorder or panic disorder, those who chose to continue other psychotropic medication or psychotherapy.
Interventions	Nortriptyline - bedtime dose of 3 capsules given as soon as possible after birth, ideally within 24 hours, increase daily for 1 week postpartum, 20, 30, 40, 50, 60, 70mg/day and 75 mg to day 21. Serum level from day 14 determined dose from then on to achieve 50-150ng/ml serum level. Dose tapered from week 17 at 33% per week and discontinued at week 20 postpartum.
Outcomes	1. 6/26 women taking nortriptyline had recurrences (0.23, 95% CI 0.09, 0.44) and 6/25 women taking placebo had recurrences (0.24, 95% CI 0.09, 0.45), Fisher's exact p = 1.00.<br>2. There was no difference in time to recurrence between the nortriptyline and placebo treatments; exact log rank =<0.001, exact p = 0.83.<br>3. There was no difference in time to recurrence between nortriptyline and placebo when stratified by presence of non-post partum depression episode (exact log rank =<0.001, exact p = 0.83).
Notes	Power calculation reported as 'power statements for proportion recurring were done with exact binomial probabilities'. No calculations provided.
Allocation concealment	B

Study	Wisner 2004
Methods	Randomised controlled trial. Method of randomisation: randomised in a 2:1 ratio for sertraline v.s placebo; other details not given. 38 eligible women screened, 25 consented to participate, 3 withdrew after randomisation to sertraline. Randomised 2:1, sertraline:<br>placebo. Outcome assessed by blind assessor.
Participants	Recruited from women's psychiatric out patient department, USA. Pregnant women age 21-45. Inclusion criteria: healthy with normal thyroid studies and blood count,<br><35 weeks gestation at recruitment,<br>at least 1 episode of postpartum major depression fulfilling DSM IV.<br>Criteria within 5 years of study enrollment.<br>Exclusion criteria: depression during index pregnancy,<br>use of psychotherapy or psychotropic medication after 1st trimester,<br>other Axis 1 diagnoses (except general anxiety or panic disorder), antisocial or borderline personality disorder, or had psychosis or bipolar disorder.

Copyright © 2006 The Cochrane Collaboration. Published by John Wiley & Sons, Ltd

Characteristics of included studies (*Continued*)

Interventions	Sertraline - single post breakfast dose in 2 identical opaque gelatin capsules.
Outcomes	1. Fewer recurrences in women treated with sertraline (1/14) (0.07, 95%CI 0.00, 0.34) than placebo (4/8) (0.50, 95%CI, 0.16, 0.84) (Fishers exact p=0.04).<br>2. Time to recurrence was also quicker in the placebo group (exact Wilcoxon Gehan p=0.02). The observed hazard ratio was 9.09 (95%CI, 0.98, 88.3).
Notes	No power calculation reported
Allocation concealment	B

ANALYSES

Comparison 01. Nortriptyline versus placebo

Outcome title	No. of studies	No. of participants	Statistical method	Effect size
01 Recurrence of postpartum MDD	1	51	Relative Risk (Fixed) 95% CI	0.96 [0.36, 2.59]

Comparison 02. Sertraline versus placebo

Outcome title	No. of studies	No. of participants	Statistical method	Effect size
01 Recurrence of postpartum MDD	1	22	Relative Risk (Fixed) 95% CI	0.14 [0.02, 1.07]

INDEX TERMS

Medical Subject Headings (MeSH)

Antidepressive Agents [*therapeutic use]; Depression, Postpartum [*prevention & control]; Nortriptyline [therapeutic use]; Randomized Controlled Trials; Sertraline [therapeutic use]

MeSH check words

Female; Humans

COVER SHEET

**Title**	Antidepressant prevention of postnatal depression
**Authors**	Howard LM, Hoffbrand S, Henshaw C, Boath L, Bradley E
**Contribution of author(s)**	All five authors developed the protocol. Louise Howard is the lead investigator. LH conceived and designed the review, and coordinated the review; she entered data into Rev Man, analysed and interpreted data, and wrote the review. SH developed the search strategy, entered data into Rev Man, and interpreted data. CH and E Boath undertook searches, retrieval of papers, screened retrieved papers against inclusion criteria, appraised quality of papers and abstract data from papers, contacted authors for additional information, and obtained and screened data on unpublished studies. E Bradley analysed and interpreted data, and wrote the discussion and synopsis with E Boath.
**Issue protocol first published**	2003/3
**Review first published**	2005/2
**Date of most recent amendment**	25 February 2005

Copyright © 2006 The Cochrane Collaboration. Published by John Wiley & Sons, Ltd

**Date of most recent SUBSTANTIVE amendment**	31 January 2005
**What's New**	Information not supplied by author
**Date new studies sought but none found**	Information not supplied by author
**Date new studies found but not yet included/excluded**	Information not supplied by author
**Date new studies found and included/excluded**	Information not supplied by author
**Date authors' conclusions section amended**	Information not supplied by author
**Contact address**	Dr Louise Howard<br>Senior Lecturer<br>Box PO29<br>Institute of Psychiatry<br>De Crespigny Park<br>Denmark Hill<br>London<br>SE5 8AF<br>UK<br>E-mail: spjelmh@iop.kcl.ac.uk<br>Tel: +44 20 7848 0735<br>Fax: +44 20 7277 1462
**DOI**	10.1002/14651858.CD004363.pub2
**Cochrane Library number**	CD004363
**Editorial group**	Cochrane Depression, Anxiety and Neurosis Group
**Editorial group code**	HM-DEPRESSN

GRAPHS AND OTHER TABLES

Analysis 01.01. Comparison 01 Nortriptyline versus placebo, Outcome 01 Recurrence of postpartum MDD

Review: Antidepressant prevention of postnatal depression

Comparison: 01 Nortriptyline versus placebo

Outcome: 01 Recurrence of postpartum MDD

Study	Nortriptyline n/N	Placebo n/N	Relative Risk (Fixed) 95% CI	Weight (%)	Relative Risk (Fixed) 95% CI
Wisner 2001	6/26	6/25		100.0	0.96 [0.36, 2.59]
Total (95% CI)	26	25		100.0	0.96 [0.36, 2.59]

Total events: 6 (Nortriptyline), 6 (Placebo)

Test for heterogeneity: not applicable

Test for overall effect z=0.08 p=0.9

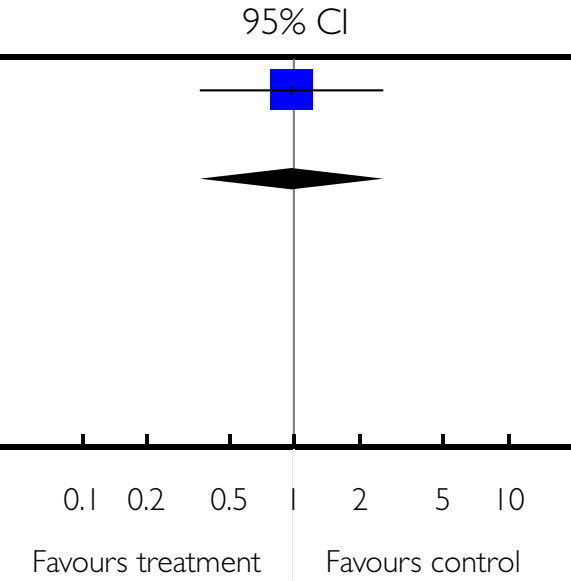

Copyright © 2006 The Cochrane Collaboration. Published by John Wiley & Sons, Ltd

Analysis 02.01. Comparison 02 Sertraline versus placebo, Outcome 01 Recurrence of postpartum MDD

Review: Antidepressant prevention of postnatal depression

Comparison: 02 Sertraline versus placebo

Outcome: 01 Recurrence of postpartum MDD

Study	Sertraline n/N	Placebo n/N	Relative Risk (Fixed) 95% CI	Weight (%)	Relative Risk (Fixed) 95% CI
Wisner 2004	1/14	4/8		100.0	0.14 [0.02, 1.07]
Total (95% CI)	14	8		100.0	0.14 [0.02, 1.07]

Total events: 1 (Sertraline), 4 (Placebo)

Test for heterogeneity: not applicable

Test for overall effect z=1.90 p=0.06

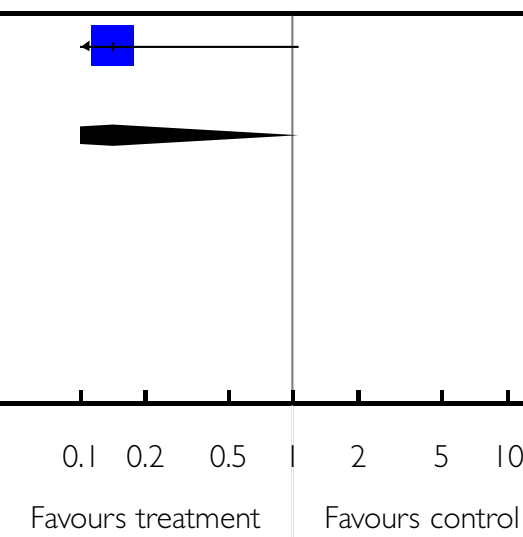

Copyright © 2006 The Cochrane Collaboration. Published by John Wiley & Sons, Ltd
